# Effect of Pulse Rate and Polarity on the Sensitivity of Auditory Brainstem and Cochlear Implant Users to Electrical Stimulation

**DOI:** 10.1007/s10162-015-0530-z

**Published:** 2015-07-03

**Authors:** Robert P. Carlyon, John M. Deeks, Colette M. McKay

**Affiliations:** MRC Cognition & Brain Sciences Unit, 15 Chaucer Rd., Cambridge, England; Audiology & Deafness Research Group, School of Psychological Sciences, University of Manchester, Manchester, M13 9PL England; The Bionics Institute of Australia, 384 Albert St, East Melbourne, 3002 Australia

**Keywords:** auditory brainstem implants, cochlear implants

## Abstract

To further understand the response of the human brainstem to electrical stimulation, a series of experiments compared the effect of pulse rate and polarity on detection thresholds between auditory brainstem implant (ABI) and cochlear implant (CI) patients. Experiment 1 showed that for 400-ms pulse trains, ABI users’ thresholds dropped by about 2 dB as pulse rate was increased from 71 to 500 pps, but only by an average of 0.6 dB as rate was increased further to 3500 pps. This latter decrease was much smaller than the 7.7-dB observed for CI users. A similar result was obtained for pulse trains with a 40-ms duration. Furthermore, experiment 2 showed that the threshold difference between 500- and 3500-pps pulse trains remained much smaller for ABI than for CI users, even for durations as short as 2 ms, indicating the effect of a fast-acting mechanism. Experiment 3 showed that ABI users’ thresholds were lower for alternating-polarity than for fixed-polarity pulse trains, and that this difference was greater at 3500 pps than at 500 pps, consistent with the effect of pulse rate on ABI users’ thresholds being influenced by charge interactions between successive biphasic pulses. Experiment 4 compared thresholds and loudness between trains of asymmetric pulses of opposite polarity, in monopolar mode, and showed that in both cases less current was needed when the anodic, rather than the cathodic, current was concentrated into a short time interval. This finding is similar to that previously observed for CI users and is consistent with ABI users being more sensitive to anodic than cathodic current. We argue that our results constrain potential explanations for the differences in the perception of electrical stimulation by CI and ABI users, and have potential implications for future ABI stimulation strategies.

## INTRODUCTION

Auditory brainstem implants (ABIs) can restore hearing to deaf patients for whom a cochlear implant (CI) is unsuitable. The most common reason for implanting an ABI is damage to the auditory nerve, for example as a result of tumour removal in patients with neurofibromatosis type II (‘NF2’). In contrast to CI users, open-set speech perception for ABI users with NF2 is usually very poor, although better overall outcomes have been reported for patients with non-tumour aetiologies (Colletti and Shannon 2005) and for a minority of NF2 patients (Colletti et al. 2012; Matthies et al. 2014). Differences between ABI and CI users have also been observed in the small number of psychophysical studies that have studied the basic effects of brainstem stimulation (Shannon and Otto 1990; Zeng and Shannon 1992, 1994; Colletti and Shannon 2005; Long et al. 2005; Lim et al. 2008; McKay et al. 2015). For example, whereas it is not unusual for CI users to be able to discriminate between every adjacent pair of electrodes, stimulation of the different electrodes of an ABI typically yields only three or four discriminable pitches (Long et al. 2005).

The present study addresses two fundamental questions concerning how the human brainstem responds to electrical stimulation. The first concerns the way in which the effects of successive pulses combine to influence the listener’s perception, and examines the effect of pulse rate and duration on detection thresholds. The second addresses the question of whether the human brainstem is primarily sensitive to anodic or to cathodic current, and, hence, on which part of each biphasic pulse is primarily responsible for the detection and loudness of a pulse train. The answers to both questions not only provide insight into the basic mechanisms underlying the perception of electrical stimulation of the human brainstem but also have potential applications for ABI processing strategies and fitting.

For CI users, it is well known that thresholds and loudness levels drop as pulse rate is increased from a few pulses per second (pps) up to several thousand pps. For pulse durations that are typically used clinically (below a few hundred microseconds per phase), the dB change in current level for a doubling of pulse rate is greatest for rates above about 100–300 pps (Shannon 1985; McKay and McDermott 1998). There is some evidence, however, that the effect of pulse rate on threshold is different for ABI users. Lim et al (2008) studied the effects of pulse rate on thresholds in users of the auditory midbrain implant and included data from three ABI users, each representative of a group showing a different pattern of results: one of these was similar to CI users, one showed no effect of pulse rate, and one showed a threshold drop up to 100–200 pps, with no further drop at higher rates. Shannon (2011) presented some unpublished data that, despite some marked individual differences, showed that thresholds for the majority of ABI patients dropped with increases in rate up to a breakpoint of 200–300 pps, above which the rate vs. threshold function was flat. Because the stimulus duration is usually held constant at all pulse rates, this suggests that substantial increases in the number of pulses applied to the brainstem had a negligible effect on thresholds. Our first three experiments confirmed this striking finding and investigated the mechanisms responsible for the very different effect of pulse rate on thresholds for ABI versus CI users.

At least three general classes of phenomenon may contribute to the drop in threshold with increasing pulse rate for CI users. First, for rates below a few hundred pps, individual auditory nerve (AN) fibres may fire on every pulse so that the firing rate per fibre will increase with pulse rate and contribute to the detection of the stimulus. Second, even when an individual fibre is in a refractory state following one pulse, a subsequent pulse may elicit an action potential (‘spike’) on other fibres. Hence an increase in the pulse rate may increase the firing rate of the ensemble of auditory nerve fibres, even at rates above that to which individual fibres can entrain. This idea is reminiscent of the ‘volley principle’ (Wever 1949) that was initially proposed to explain the temporal coding of pitch in acoustic hearing for frequencies higher than the saturation rate of individual AN fibres. Third, even when a pulse does not cause a fibre to fire, it may increase the probability of that fibre firing to a subsequent pulse. This facilitation has been described by Middlebrooks (2004) in terms of an incomplete recovery of the partial depolarisation of the nerve membrane in response to a preceding pulse. He measured the response of neurons in the auditory cortex of anaesthetised guinea pigs in response to electrical stimulation of the AN and reported that thresholds dropped steeply with increases in pulse rate above, but not below, 1000 pps, consistent with a mechanism based on partial depolarisation. Middlebrooks (2004) assumed that the effect of increasing pulse rate at lower rates, observed in human CI users, reflected processes not revealed by his recordings. More detailed accounts of how facilitation might occur have been proposed, all of which assume that the distribution of charge across the cell membrane after the presentation of one pulse interacts with the changes in that distribution produced by a subsequent pulse (e.g., Grill and Mortimer 1999; Cartee et al. 2006).

There are also several different mechanisms by which the effect of pulse rate on threshold might be reduced for ABI users. Longer refractory periods could reduce the maximum pulse rate to which cochlear nucleus (CN) neurons entrain. A reduction in the number of excitable neurons could reduce the extent to which the effects of increasing pulse rate is shared by multiple neurons: *In extremis*, if there were only a single neuron available, then the effect of pulse rate would be limited by the saturation rate of that neuron. A reduction in the interaction between successive pulses at the level of the cell membrane could also lead to reduced effects of increasing pulse rate at high rates. All of these mechanisms would be expected to operate over a short time scale and to reduce the effects of pulse rate even for very short stimuli. Another phenomenon that could mediate the effect of pulse rate is adaptation. Zhang et al (2007) recorded the response of cat AN fibers to trains of electrical pulses of a range of rates; neurons differed in the amount of adaptation, with fewer showing substantial amounts of adaptation for 250-pps pulse trains than at higher pulse rates. They modelled the adaptation using two time constants, which they described as roughly similar to the ‘rapid’ and ‘short-term’ adaptation observed with acoustic stimulation. The rapid component had a time constant of 8 ms. The short-term component had a much longer time constant of 80 ms. A greater dependence of rapid- or short-term adaptation on stimulation rate in the brainstem could reduce the effect of pulse rate on ABI thresholds, as the reduction in the response to the end of the stimulus could counteract any beneficial effects of the increased rate early on. Evidence for greater adaptation in more central structures comes from a study of auditory midbrain implant (AMI) users, who show only a small effect of pulse rate on thresholds above about 200 pps (Lim et al. 2008; McKay et al. 2013). McKay et al (2013) showed that AMI users’ thresholds decreased with increases in the duration of the pulse train up to a certain value, after which it was constant, and this asymptotic duration decreased with increasing pulse rate.

Our first three experiments examine the time course of the mechanism(s) responsible for the different effects of pulse rate on threshold in ABI and CI users and aim to shed light on the possible explanations for this difference. Specifically, we investigate whether the small effect of pulse rate on thresholds could be due to higher rates producing greater adaptation of neural responses, and study the time course of any likely adaptation. We also investigate charge interactions at the level of the cell membrane by comparing thresholds for fixed and alternating polarity pulse trains. Such charge interactions are, as discussed above, one proposed reason for why thresholds drop with increasing rate in CI users, and so it was important to determine the extent to which such interactions occur for ABI users. These three experiments show that (a) the small effect of pulse rate on ABI users’ thresholds occurs even for very short-duration stimuli and cannot be due to long-term adaptation, (b) short-term adaptation with a time constant below 10 ms may contribute to the difference, and (c) at high pulse rates, successive pulses do interact at the level of the cell membrane, and that these interactions are at least as large for ABI as for CI users. This last finding makes it unlikely that the small effect of pulse rate for ABI users is due to successive pulses not interacting at the cell membrane.

In order to provide further information on the stimulation of the cell membrane by electrical pulse trains, a fourth experiment investigated which part or parts of each biphasic pulse contributes to detection. All previous psychophysical studies of ABI stimulation have used symmetric waveforms, which, in recent years, have been restricted to symmetric biphasic pulses, and it is therefore not known which phase (or phases) of the pulse is responsible for stimulating the brainstem. Research with CI users has used asymmetric pulses to demonstrate that they are preferentially sensitive to anodic stimulation, such that, in monopolar mode, less current is needed to achieve a given loudness when the short high-amplitude phase of an asymmetric pulse is anodic compared to when it is cathodic (Macherey et al. 2006; Macherey et al. 2008; Carlyon et al. 2013). Here, we show that a similar effect occurs for ABI stimulation, suggesting that the brainstem is primarily excited by the anodic phase of each biphasic pulse.

## EXPERIMENT 1

### Rationale

If, for ABI users, the effect of increasing pulse rate is counteracted by greater adaptation at high rates, the dependence of threshold on pulse rate should be greater at short durations. That is, at long but not at short durations, the effect of increasing the number of pulses may be counteracted by greater adaptation at the higher pulse rate. Experiment 1 therefore measured thresholds at three pulse rates and for signal durations of 40 and 400 ms. These two durations were chosen so as to test the possible effect of adaptation having a time constant longer than about 40 ms, such as the short-term adaptation reported for CI stimulation of the cat auditory nerve by Zhang et al (2007).

### Methods

Subjects for all experiments reported in this article were selected from five users of the nucleus CI and eight users of the nucleus ABI, both manufactured by Cochlear Ltd. All except subject ABI 2, took part in experiment 1. Their clinical details are shown in Table 1. All provided informed consent for this research project, which was approved by the National Research Ethics Services for the East and North West of England. All of the CI users and subjects ABI 1 and 2 were implanted at Addenbrookes hospital, Cambridge, whereas ABI subjects 3 to 8 were implanted at Manchester Royal Infirmary.

**TABLE 1:** Details of the ABI and CI users. Each column shows, from left to right, the subject’s age, aetiology, duration of deafness (years), implant device, electrode tested, pulse width (μs) and inter-phase gap (μs). The CI users and subjects ABI 1 and ABI 2 were implanted at Addenbrookes hospital, Cambridge, whereas the ABI subjects 3 to 8 were implanted at Manchester Royal Infirmary

Subject	Age	Aetiology	Dur. deaf	Device	Electrode	pw, ipg
ABI 1	20	NF2	0	ABI	18	101, 45
ABI 2	31	NF2	0	ABI	7,2	50, 45
ABI 3	21	NF2	1	ABI	14	50, 8
ABI 4	49	NF2	2	ABI	10	50, 8
ABI 5	75	Bilat VS^a^	0	ABI	12	25, 8
ABI 6	27	NF2	0	ABI	8	25, 8
ABI 7	32	NF2	10	ABI	6	101, 8
ABI 8	49	NF2	0	ABI	11	101, 8
CI 1	80	Unknown	5	CI 24 M	11	45, 8
CI 2	81	Otosclerosis	22	CI 24 M	17	45, 8
CI 3	65	CSOM	10	CI 24 M	11	45, 8
CI 4	60	Unknown	30	CI 24 M	17	45, 8
CI 5	71	Unknown	20	Freedom	17	45, 8

*NF2* neurofibromatosis type II, *Bilat VS* bilateral vestibular schwannoma, *CSOM* chronic suppurative otitis Media

^a^NF2 not confirmed

Stimuli were 40- or 400-ms trains of symmetric biphasic pulses presented at a rate of 71, 500 or 3500 pps; for the 71-pps pulse train, the shorter stimulus contained only three pulses and so the total duration was actually 28.17 ms. The first phase of each pulse was always cathodic. Stimulation was applied in monopolar (‘MP1 + 2’) mode to a single electrode, shown in Table 1, for each subject. Note that for subject ABI 2, who did not take part in experiment 1, stimuli were presented in bipolar mode, as in her clinical map. For ABI users, the electrode was chosen to be one that was used in the patient’s clinical map. For CI users, an electrode near the middle of the array (either E11 or E17) was chosen. The phase duration and inter-phase gap were 45 and 8 μs, respectively, for the CI users, and the same as that used in the clinical map for each ABI user, as shown in Table 1. All stimuli were checked using a test implant and a digital storage oscilloscope; note that the Nucleus CI and ABI use identical electronics, the only substantive difference being in the electrode array. Stimuli were presented using the NIC2 research interface controlled by a Matlab program. The NIC2 software and research hardware were provided by Cochlear Ltd.

For each subject and each pulse rate, the current level of a 400-ms pulse train was raised gradually from zero until it was judged to be a comfortable listening level. This level, minus 5 current units (‘CUs’, each equal to 0.17 dB), defined the starting point for the adaptive procedure for each rate. Thresholds were then measured using an adaptive procedure that converged on the 71 % point of the psychometric function. Each trial consisted of two intervals, one of which contained the signal. The subject indicated the signal interval by clicking on a virtual response button on a computer screen; correct answer feedback was provided after every trial. Signal level was decreased after every two consecutive correct answers and increased after every incorrect answer. The change from increasing to decreasing level or vice versa defined a turnpoint, and each run continued until eight turnpoints had been completed. The last six turnpoints were averaged and used to represent the run. The step size was four CUs for the first two turnpoints and one CU thereafter. At least two runs were obtained per condition; any time remaining at the end of a session was devoted to testing a third run for some conditions, starting with the condition showing the largest standard deviation after two runs. The average standard deviation across runs for all subjects and conditions in experiment 1 was 0.29 dB. The thresholds reported were always calculated from the mean of every run tested for a given condition.

### Results

**FIG. 1:**
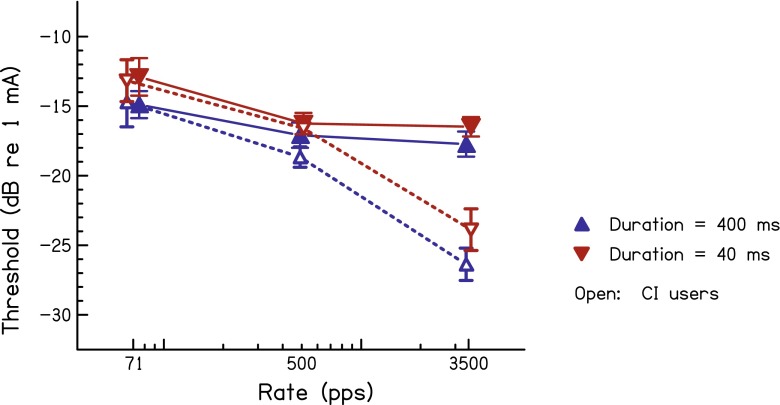
Thresholds as a function of pulse rate averaged across ABI users (*filled symbols*) and CI users (*open symbols*). Data obtained with a signal duration of 400 ms are shown in *blue* (*upward triangles*), whereas those obtained with a 40-ms duration are shown in *red* (*downward triangles*). *Error bars* show standard deviations calculated so as to remove across-listener differences in overall performance. Data points are shifted horizontally by a small amount for clarity.

Thresholds, averaged across subjects for each device type, are shown in Figure 1. Consider first the data obtained at the 400-ms duration, shown by the blue upward-pointing triangles. It is clear that the effect of pulse rate on threshold is very different for the two groups. Thresholds for CI users (open symbols) dropped by an average of 3.9 dB from 71 to 500 pps, and then showed a larger drop of 7.7 dB from 500 to 3500 pps. In contrast, thresholds for ABI users (filled symbols) dropped by 2.2 dB from 71 to 500 pps but by only 0.6 dB from 500 to 3500 pps. Importantly, the results for a 40-ms duration (red, downward-pointing triangles) are almost identical, except that thresholds are slightly higher overall. All of these trends were confirmed by an ANOVA with duration and pulse rate as within-subject factors and device type as a between-subject factor. The main effect of pulse rate was highly significant (*F*_(2,20)_ = 99.3, *P* < 0.001) and that of duration was marginally significant (*F*_(1,10)_ = 4.76, *P* = 0.054). The interaction between device and pulse rate was highly significant (*F*_(2,20)_ = 36.34, *P* < 0.001). Duration did not interact significantly with rate, and the three-way duration × device × rate interaction was also not significant.

The individual data are shown in Table 2. All of the CI users show the same trend as in the average data, with a larger threshold drop as rate is increased from 500 to 3500 pps than from 71 to 500 pps. Most ABI users also show the same trend as in the average data for that group; the threshold drop from 71 to 500 pps is larger than the small or absent drop from 500 to 3500 pps. Minor exceptions are listener ABI 3 at the 400-ms duration and ABI 4 at the 40-ms duration, where there is a small threshold drop of about the same size between 71 to 500 and 500 to 3500 pps. For the 400-ms duration, subject ABI 1 shows a pattern intermediate between that seen for the average CI and ABI data; threshold drops by 1.4 dB from 71 to 500 pps and by 3.5 dB from 500 to 3500 pps. For the 40-ms duration, her results are more similar to those of the other ABI users, with only a 0.1 dB threshold drop between 500 and 3500 pps.

**TABLE 2:** Thresholds (dB re 1 mA) as a function of pulse rate (pps) and duration (ms) for the ABI and CI users who took part in experiment 1

Subject	400 ms			40 ms		
	71	500	3500	71	500	3500
ABI 1	−7.1	−8.5	−12.0	−6.1	−7.3	−7.4
ABI 3	−15.3	−16.7	−17.2	−12.6	−16.5	−16.7
ABI 4	−17.0	−17.4	−19.2	−15.3	−16.7	−18.1
ABI 5	−14.9	−20.2	−19.8	−11.8	−19.2	−19.4
ABI 6	−16.4	−17.6	−16.8	−14.4	−17.0	−16.6
ABI 7	−18.5	−20.7	−21.0	−15.7	−19.5	−19.6
ABI 8	−15.0	−18.5	−18.1	−14.5	−17.5	−17.6
CI 1	−12.7	−15.7	−22.9	−11.4	−13.5	−19.7
CI 2	−15.6	−16.3	−23.2	−13.8	−15.8	−21.1
CI 3	−9.7	−15.7	−24.2	−8.7	−13.4	−21.3
CI 4	−16.5	−19.7	−28.3	−15.2	−18.2	−26.2
CI 5	−19.1	−25.8	−33.3	−16.7	−22.1	−31.2

Experiment 1 provided statistically significant evidence that pulse rate has a different effect on thresholds for ABI users compared to CI users (cf. Lim et al. 2008; Shannon 2011). It shows that this difference was similar at the two durations, demonstrating that the difference between the two groups is mediated by a mechanism that operates over a time course that is shorter than 40 ms. To study this time course in more detail, experiment 2 measured CI and ABI users’ thresholds at 500 and 3500 pps over an even shorter range of durations.

## EXPERIMENT 2

### Rationale

Experiment 2 measured the effect of pulse rate on thresholds for durations ranging from about 2 to 32 ms and including measurements for a single pulse. The aim was to determine whether there exists a range of short durations over which there is a substantial (or at least larger) effect of pulse rate on ABI listeners’ thresholds. If not, then we can conclude that the small effect of pulse rate for ABI users is due to a mechanism or feature of brainstem stimulation that is almost instantaneous. If there does exist such a range, then the maximum duration over which a larger effect of pulse rate is observed will inform us about the time constant of the mechanisms involved. The range of durations tested encompasses the time constant of the rapid component of the adaptation described for cat auditory nerve stimulation by Zhang et al (2007).

### Methods

The subjects were five ABI users (numbers 1, 3, 6, 7 and 8) and four CI users (numbers 1, 2, 3 and 4). The phase durations and inter-phase gaps were the same as in experiment 1. Because the phase duration was 45 μs for the CI users and between 25 and 101 μs for the ABI users, two of the CI users were additionally tested with a phase duration of 100 μs.

**FIG. 2:**
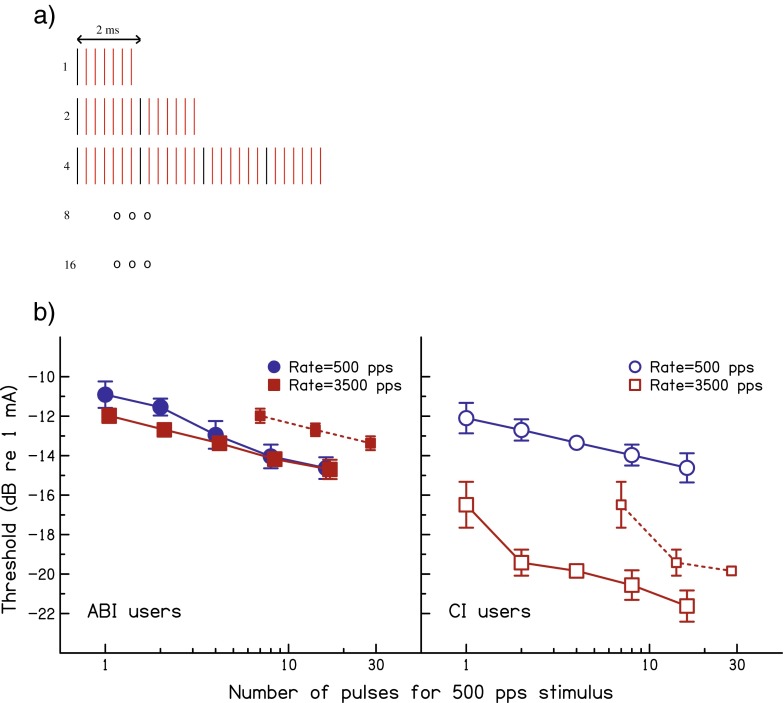
**A** Schematic of the conditions of experiment 2. Each pulse is shown as a vertical line. The 500-pps condition is represented by the *black lines* whereas the 3500-pps condition is represented by *all lines*. Only the stimuli for NumPulse500 = 1, 2 and 4 are shown for clarity. **B** Thresholds from experiment 2 averaged across ABI users (*filled symbols*) and CI users (*open symbols*). Data for pulse rates of 500 and 3500 pps are shown by *blue circles* and *red squares*, respectively. *Error bars* show standard deviations calculated so as to remove across-listener differences in overall performance. The *abscissa* shows the number of pulses in the corresponding 500-ms stimulus (‘NumPulse500’); hence, the actual number of pulses in the 3500-pps stimuli was seven times these values. The *red dashed lines* with smaller symbols show the 3500-pps data re-plotted in terms of the actual number of pulses in the stimulus for the three shortest durations.

A schematic of the stimuli is shown in Figure 2A. In the 500-pps condition, shown by the black lines, the signal consisted of 1, 2, 4, 8 or 16 pulses with an inter-pulse interval of 2 ms. The 3500-pps condition is shown by the combination of the red and black lines; it was generated by following every pulse in the 500-pps condition by six pulses each separated from its neighbours by 0.285 ms (the reciprocal of 3500 pps). The durations of the 3500-pps stimuli were therefore 1.71, 3.71, 7.71, 15.71 and 31.71 ms, respectively, in addition to the duration of a single pulse. In all other respects, the method and stimuli were the same as in experiment 1.

### Results

The solid lines in Figure 2B show thresholds, averaged across the subjects in each group, as a function of the number of pulses in the 500-pps stimulus. Data for the 500-pps stimulus are shown by the blue circles, with the corresponding 3500-pps condition shown as red squares. At each horizontal location, then the number of pulses in the 3500-pps stimulus is seven times larger than that for the corresponding 500-pps stimulus. Despite this sevenfold increase in charge, thresholds plotted in this way for the 500 and 3500-pps pulse trains are very similar for the ABI listeners (left-hand panel), whereas they differ by a substantially larger amount for the CI users (right-hand panel). This is true even at the shortest duration, where the comparison is between a single pulse and seven pulses and where thresholds in the two conditions differ by 4.3 and 1.1 dB for the CI and ABI users, respectively. The differing effects of pulse rate for the two groups of users were confirmed by a two-way ANOVA, with rate and ‘number of pulses at 500 pps (henceforth NumPulse500)’ as within-subject factors and device type as a between-subject factor. Both the main effect of pulse rate and its interaction with device type were highly significant (main effect: *F*_(1,7)_ = 104.67; *P* < 0.001; interaction: *F*_(1,7)_ = 72.77, *P* < 0.001). The only other significant effects were the interaction between rate and NumPulse500 (*F*_(4,28)_ = 3.14, *P* = 0.03) and the three-way interaction between these two factors and device type (*F*_(4,28)_ = 8.32, *P* = 0.001). Inspection of Figure 2B suggests that both of these effects are due to the large drop in threshold between the two shortest durations at 3500 pps for the CI users, which was larger than that for the ABI users and larger than that at 500 pps for either group.

A potential factor influencing thresholds at short durations is the length of the ‘temporal window’ over which pulses are integrated (Plack and Moore 1990), which could differ between excitation of the AN and of the brainstem, as has been shown to be the case when comparing excitation of the AN and the midbrain (McKay et al.^ 2013^). This could affect the observed influence of pulse rate because, given the way we have presented and analysed the data, each 3500-pps stimulus is slightly longer than its 500-pps counterpart. However, the experiment includes two conditions—two pulses at 500 pps and a single pulse followed by six further pulses at 3500 pps—with very similar durations. The difference between these two conditions (first red and second blue symbol from the left in each panel of Fig. 2B) corresponds to the effect of filling in the gap between two pulses, separated by 2 ms, with five pulses 0.285 ms apart. Here, the difference is also much larger for the CI users (3.8 dB) than for the ABI users (0.4 dB).

Further insight can be obtained by inspecting the dashed red lines in Figure 2B, which show the first three points of the 3500-pps data plotted in terms of the actual number of pulses, rather than in terms of NumPulse500. Comparison with the 500-pps data (blue circles) reveals that, for an equal number of pulses, ABI users’ thresholds are always higher at 3500 pps than at 500 pps. Because, for an equal number of pulses, the stimulus is shorter for the 3500-pps than for the 500-pps rate, this difference is unlikely to be due to some pulses falling outside of a central temporal window. Note also that the opposite trend occurs for CI users, who always show lower thresholds for a given number of pulses at the 3500-pps than at the 500-pps rate.

The fact that the effect of pulse rate is smaller for the ABI than for the CI users even at the shortest durations suggests that the difference is strongly influenced by a mechanism that operates over a very short time course. However, it is also apparent from Figure 2B that, for the ABI listeners, the effect of pulse rate is slightly larger at the shortest two durations. This was confirmed by a two-way ANOVA of just the ABI data, which not only revealed significant effects of NumPulse500 and of rate but also a two-way interaction (NumPulse500: *F*_(4,16)_ = 34.6; *P* < 0.001. Rate: *F*_(1,4)_ = 11.83; *P* = 0.026. Interaction: *F*_(4,16)_ = 6.43; *P* < 0.005). This is consistent with an additional contribution from a mechanism having a slightly longer time course. The results provide no evidence that the effects of pulse rate on threshold are mediated by more extended processes having a time constant substantially longer than about 7–8 ms, corresponding to the third point from the left in each part of Figure 2B. Possible physiological bases for the effects described here are considered in the ‘Discussion’.

**FIG. 3:**
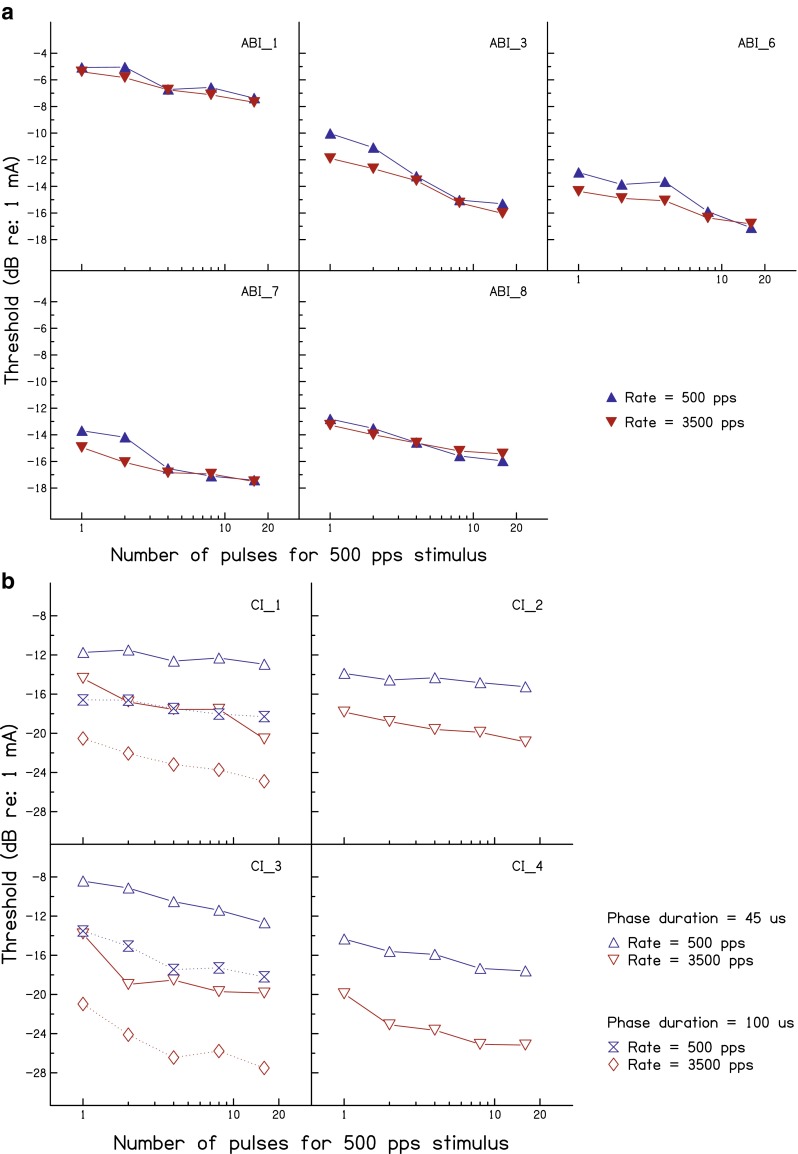
Parts **A** and **B** show the results of experiment 2 for individual ABI and CI listeners, respectively. The format of the plots is the same as for the mean data shown in Fig. 2, except that no error bars are shown (because thresholds were usually obtained only from the mean of two runs).

Data for the individual ABI and CI users are shown in Figure 3A, B, respectively. Three of the ABI users show a slightly larger effect of pulse rate at the two (three for ABI 6) shortest durations than at the longer durations. However, even for these listeners and at these short durations, the effect of pulse rate was smaller than 2 dB compared to an average difference of 4 dB for the CI users (Fig. 3B). The pattern of results did not differ substantially among the CI users. Furthermore, phase duration did not have a substantial effect on the pattern of results for the two subjects tested additionally with the longer phase duration of 100 μs (symbols with dotted lines).

## EXPERIMENT 3

### Rationale

As noted in the ‘Introduction’, it has been suggested that charge interactions at the level of the cell membrane may contribute to the fact that, for pulse rates above a few hundred pps, further increases in rate reduce thresholds substantially for CI users. An absence or reduction in such charge interactions in ABI users could then potentially explain the smaller effect of pulse rate on thresholds in this group. For example, if the cell membrane returned completely to its resting potential after every pulse then, presumably, the partial depolarisation effect proposed by Middlebrooks to account for the beneficial effect of high pulse rates in CI users would not apply to ABI users.

**FIG. 4:**
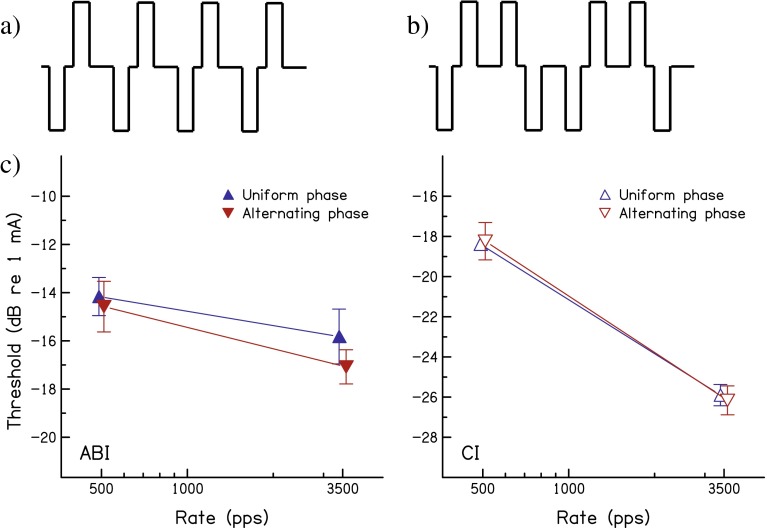
**A** and **B**: schematic of a fixed- polarity and an alternating- polarity pulse train. The left and right panels of part **C** show thresholds for the two pulse rates and polarity conditions, averaged across the ABI and CI listeners respectively, as a function of pulse rate. *Error bars* show standard deviations calculated so as to remove across-listener differences in overall performance. For clarity, there is a small horizontal offset between symbols for the same pulse rate and subject group.

To determine whether charge interaction between successive pulses occurs for ABI users, experiment 3 examined the effect of alternating the leading polarity from pulse to pulse. Increasing the pulse rate reduces the time between the second phase of one pulse and the first phase of the next, and, for the stimuli of experiments 1 and 2, these two phases have opposite polarity (Fig. 4A). In contrast, when the leading polarity alternates from pulse to pulse, the adjacent phases of neighbouring pulses have the same polarity (Fig. 4B).

### Method

Thresholds were obtained for fixed- and alternating-polarity conditions and for pulse rates of 500 and 3500 pps. The first phase of each pulse was cathodic for all pulses in the fixed-polarity condition and for every odd-numbered pulse in the alternating-polarity condition. Four ABI and four CI users, listed in Table 3, took part. Their data for the fixed-polarity condition were taken from the 400-ms condition of experiment 1, except for subject ABI 2 who did not take part in that experiment. The stimulus duration was always 400 ms. In all other respects, the method and procedure were the same as in experiment 1.

**TABLE 3:** Thresholds (dB re 1 mA) for fixed- and alternating polarity pulse trains, and the difference (F-A) between these thresholds, for the ABI and CI users who took part in experiment 3. The phase duration (PD) and inter-phase gap (IPG) used for each subject are shown in μs

	PD, IPG	500 pps			3500 pps		
		Fixed	Alt	F-A	Fixed	Alt	F-A
ABI 1	101, 45	−8.5	−8.3	−0.2	−12.0	−12.5	0.5
ABI 2	50, 45	−13.0	−13.6	0.6	−16.0	−17.3	1.4
ABI 3	50, 8	−16.7	−17.1	0.4	−17.2	−18.4	1.2
ABI 8	101, 8	−18.5	−19.3	0.8	−18.1	−20.1	2.0
CI 1	45, 8	−15.7	−15.2	−0.5	−22.9	−23.5	0.6
CI 2	45, 8	−16.3	−17.3	1.0	−23.2	−22.8	−0.4
CI 3	45, 8	−15.7	−14.9	−0.8	−24.2	−24.0	−0.2
CI 5	45, 8	−25.8	−25.6	−0.2	−33.3	−34.3	1.0

### Results

The left and right panels of Figure 4C show thresholds for the two pulse rates and polarity conditions averaged across the ABI and CI listeners, respectively. For the ABI users, thresholds were lower in the alternating-polarity than in the fixed-polarity condition by 0.41 dB at 500 pps and by 1.25 dB at 3500 pps. A two-way (rate × polarity) repeated-measures ANOVA revealed a significant effect of polarity (*F*_(1,3)_ = 10.93; *P* = 0.046) and a significant interaction (*F*_(1,3)_ = 52.85, *P* = 0.005). Hence, for these listeners, switching to alternating polarity produces a significant threshold reduction that is larger at the higher rate. Neither the effect of alternating polarity nor its interaction with pulse rate was significant for the CI users. A combined (group × rate × polarity) ANOVA revealed a borderline significant interaction between group and polarity (*F*_(1,6)_ = 5.91; *P* = 0.051), providing some evidence that the threshold difference between fixed and alternating polarity was larger for ABI users than for CI users. The ANOVA also revealed main effects of polarity (alt vs fixed: *F*_(1,6)_ = 7.70; *P* = 0.032) and of pulse rate (rate *F*_(1,6)_ = 85.9, *P* < 0.001). Pulse rate had a larger effect for the CI than for the ABI users, as revealed by a significant interaction between pulse rate and group (*F*_(1,6)_ = 28.43, *P* = 0.002).

The results of experiment 3 revealed the effects of significant charge interactions between successive pulses for the ABI users. Strictly speaking, it is possible that this group’s lower thresholds for the alternating-polarity condition could be simply due to the presence of some anodic-leading pulses, if thresholds for those pulses were lower than for the cathodic-leading pulses. However, this would not explain why the difference between ABI users’ fixed and alternating-polarity thresholds was greater at 3500 pps than at 500 pps.

The results of individual subjects are shown in Table 3. For the ABI users, two important outcomes from the ANOVAs on the combined data are true for every subject: thresholds were higher, averaged across rates, for fixed than for alternating polarity pulse trains, and this difference was larger at 3500 pps than at 500 pps. This suggests that the statistically significant findings obtained in the ANOVAs were not unduly influenced by outliers.

It is worth noting that, although the phase duration and inter-phase gap differed between some of the ABI subjects and the CI subjects, subjects ABI 2 and ABI 3, whose stimulus parameters were closest to those used for the CI users, showed a polarity effect that was intermediate between those shown for the two other ABI subjects, for whom a longer phase duration was used. Hence, it seems unlikely that the larger effect of alternating vs fixed polarity for the ABI users, compared to the CI users, is due to the differences in stimulus parameters. Furthermore, the results clearly show that, for the stimulus parameters for which ABI users typically show only a small threshold drop between 500 and 3500 pps, there is nevertheless a significant effect of polarity on the 3500-pps thresholds, thereby indicating the presence of charge interaction.

One between-subject difference that *does* appear to co-vary with a stimulus parameter is the effect of increasing pulse rate from 500 to 3500 pps for the fixed-polarity stimuli. Subjects ABI 3 and ABI 8 showed very small threshold differences of 0.5 and −0.4 dB, respectively, as was the case for the majority of ABI users in experiment 1. Subject ABI 1, whose data are re-used here, showed a difference that was larger (3.5 dB) than for the other ABI users, although smaller than that observed for CI users. Subject ABI 2 shows a similar difference, of 3.0 dB, and these two subjects were the only ones to use the longer inter-phase gap of 45 μs. Hence, it is possible that a larger effect of pulse rate could be obtained by using even longer inter-pulse intervals. However, caution is necessary when interpreting data from just two subjects.

## EXPERIMENT 4

### Rationale

**FIG. 5:**
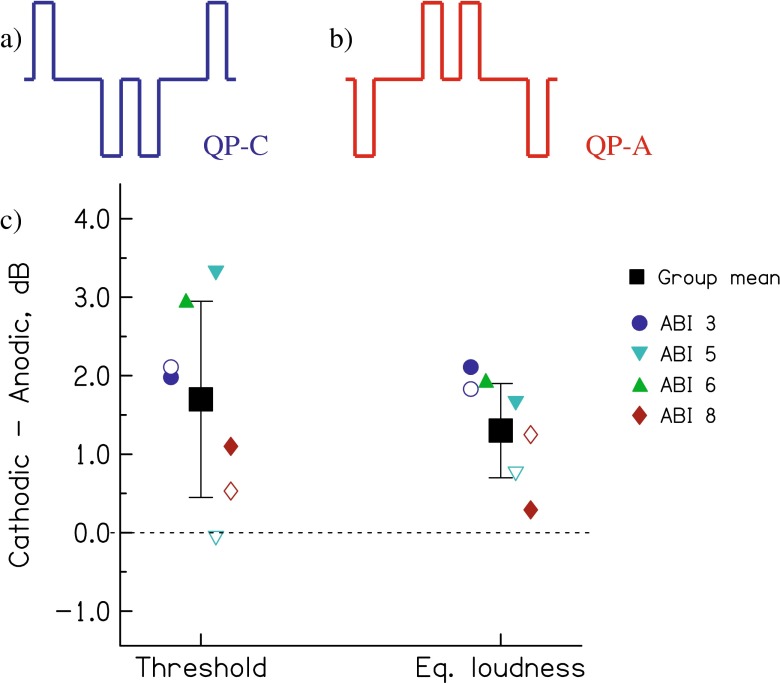
**A**, **B** Schematic of the quadraphasic pulses used in experiment 4 **A** QP-C, **B** QP-A. The inter-phase gap was 52 μs and the inter-pulse gap was 8 μs. The phase duration was 25 μs for subjects 5 and 6, 50 μs for subject 3, and 101 μs for subject 8. **C** Results of experiment 4, plotted as the difference between QP-C and QP-A threshold (*left*) and loudness (*right*) measures. Average data are shown by the *large solid squares*, with *error bars* showing the across-subject standard deviation. Each subject’s data is shown using the same symbol, which is solid for the electrode used in the previous experiments (Table 1) and, where tested, open for the ‘new’ electrode.

As noted in the ‘Introduction’, previous experiments using asymmetric pulses have shown that CI users are more sensitive to anodic than to cathodic current. Those experiments include behavioural studies of loudness, masking and pitch perception (Macherey et al. 2006; Macherey et al. 2008; Macherey et al. 2011); the conclusion that the anodic phase elicits the neural response has been confirmed by electrophysiological measures of the auditory nerve and brainstem response (Undurraga et al. 2010, 2013). Many of those studies used pseudomonophasic (‘PS’) pulses, consisting of a short, high-amplitude phase preceded by a longer low-amplitude phase of the opposite polarity. Neither the CIs nor the ABIs manufactured by Cochlear Ltd. permit the presentation of PS pulses. However, it is still possible to study polarity sensitivity by abutting two symmetric biphasic pulses with opposite leading polarity and with a long inter-phase gap. As shown in Figure 5A, B, this quadraphasic (‘QP’) pulse consists of two phases of the same polarity flanked by two single pulses of the opposite polarity. A recent CI study showed that, for equal loudness, less current is required when the central portion of the pulse is anodic than when it is cathodic (Carlyon et al. 2013). This is consistent with the findings obtained for PS pulses, where less current is needed when the short high-amplitude phase is anodic. Similar findings have been obtained with triphasic stimulation and with PS pulses in which the high-amplitude phase is presented after, rather than before, the long low-amplitude phase (Carlyon et al. 2013; Undurraga et al. 2013). In both cases, less current is needed when all of the anodic charges are concentrated in a short time period, rather than being distributed over time. Experiment 4 used QP pulses to test the polarity sensitivity of the ABI, both at threshold and at a supra-threshold level.

### Method

Four ABI listeners—numbers 3, 5, 6 and 8—took part. All were tested using the same electrode as in the previous experiments (Table 1); subjects 3, 5 and 8 were additionally tested on electrodes 4, 4 and 5, respectively. The stimuli were 400-ms 99-pps trains of QP pulses, with the central portion of each pulse either anodic (QP-A) or cathodic (QP-C). Each QP pulse consisted of two symmetric biphasic pulses, each with an inter-phase gap of 52 μs, with the two pulses separated by 8 μs (Fig. 5A, B). The phase duration was 25 μs for subjects 5 and 6, 50 μs for subject 3 and 101 μs for subject 8. Detection thresholds were measured using the same method as in experiment 1. For supra-threshold loudness matching, the QP-A and QP-C stimuli were initially adjusted to be at a maximum comfort level, with careful note made of ‘comfortable but soft’ and ‘soft’ levels. This was achieved by gradually increasing current from presentation to presentation, during which time the subject pointed to different loudness levels indicated on an 11-point scale. The loudness matching of QP-C and QP-A involved a 2-interval presentation of a fixed (reference) stimulus followed (after an inter-stimulus interval of 1 s) by an adjustable (test) stimulus. Initially, the reference stimulus was QP-A set at the comfortable-but-soft level, and the test stimulus was QP-C set to a soft level. Subjects were then asked to adjust the loudness of the test stimulus until it matched that of the reference stimulus. Subjects could increase or decrease the level of the test stimulus in steps of 1, 2 or 3 CUs, as labelled by six response buttons on a GUI; a limitation was imposed such that it could not be adjusted to a level higher than its maximum comfort level. Subjects were encouraged to ‘bracket’ their matches by increasing and decreasing the level until they were confident they heard the test stimulus as alternately louder and softer than the reference stimulus. When they were confident the test stimulus matched the reference stimulus in loudness, they pressed a button to record the final level setting. This was repeated four times, each with a slightly different initial soft level of the test stimulus. The entire procedure was then repeated with the QP-C as the reference stimulus and QP-A as the test. The matched loudness of QP-A was then taken as the comfortable-but-soft level of QP-C plus the mean level difference obtained from all eight matches. Note that we chose to perform the loudness balancing at a ‘comfortable-but-soft’ level to allow a greater degree of ‘louder’ adjustments whilst still remaining well within the maximum comfortable limits.

### Results

Figure 5C shows the difference between QP-C and QP-A threshold and loudness measures. Average data are shown by the large solid squares. Each subject’s data are shown using the same symbol, which is solid for the electrode used in the previous experiments (Table 1) and, where tested, open for the ‘new’ electrode. It can be seen that all points are above or (in one case) very close to zero, corresponding to greater sensitivity to the QP-A than to the QP-C pulses. This effect was confirmed by separate ANOVAs performed for the threshold and for the matched loudness levels, with polarity as a within-subject factor and subject as a between- subject factor. In both cases, there was a main effect of polarity (loudness: *F*_(1,3)_ = 37.43, *P* = 0.009; threshold: *F*_(1,3)_ = 11.07, *P* = 0.044). In neither case did the polarity effect interact with subject.

## DISCUSSION

### Comparison to Previous Results

Experiments 1 and 2 show that the function relating threshold to pulse rate is very different for ABI and CI listeners, and,crucially, extends this finding to a range of very short durations. Two previous studies have investigated the effect of pulse rate on thresholds for ABI users at a single duration. In a conference presentation, Shannon (2011) reported rate vs threshold functions for five NF2 ABI subjects over a wide range of rates (10–20 pps up to 1000–3000 pps, depending on the subject). For four of them, threshold dropped as rate increased up to some breakpoint, of between 100 and 200 pps, and was roughly constant at higher rates. The fifth subject showed no threshold change over the entire range between 10 and 2000 pps. A flat function was also observed for three out of the seven non-tumour ABI subjects that he described (the remainder of whom showed a pattern similar to the NF2 patients) and for two subjects with a penetrating electrode array. The duration was not given. Lim et al (2008) presented examples of three types of function that they had observed for ABI users for a 500-ms pulse train: one flat, one that reached a breakpoint at about 100 pps, and one more similar to that found for CI users. They did not indicate the relative prevalence of each pattern. Note that, with the few exceptions noted in the Results section, all of our subjects showed the same general pattern as each other, with a threshold drop from 71 to 500 pps and a smaller or absent drop from 500 to 3500 pps. As we only tested three rates, it is of course possible that the exact form of a more detailed function would differ across subjects.

There exist no previous data on the effect of pulse rate on ABI users’ thresholds at more than one duration. Shannon and Otto (1990) presented a temporal integration function averaged over 10 subjects, for whom the stimuli were either a 1000-Hz sinusoid or a 1000-pps pulse train with durations between 2 and 1000 ms. Thresholds were essentially constant for durations above about 30–40 ms. In contrast, all of our ABI users showed lower thresholds at the 400-ms than at the 40-ms duration, at all pulse rates, with the average difference being 2.0, 0.8 and 1.3 dB at 71, 500 and 3500 pps, respectively. Shannon and Otto found that the threshold drop from 2 to 30 ms was about 2 dB compared to the drops of 2.7 and 3.1 dB observed here. Hence, there is some evidence, at least over the longer range of durations, that our subjects showed more temporal integration than those in Shannon and Otto’s study. Unfortunately, basic psychophysical studies with ABI users are very scarce, with only a handful of published reports in the 25 years since Shannon and Otto’s work (Zeng and Shannon 1992, 1994; Colletti and Shannon 2005; Long et al. 2005; Lim et al. 2008; Azadpour and McKay 2014; McKay et al. 2015).

### Physiological Basis of Observed Effects

#### Pulse Rate

There are of course multiple differences between the AN and brainstem, and our measurements were psychophysical rather than physiological. Hence, it is not possible to identify the exact neural basis for the very different effects of pulse rate on detection for ABIs compared to CIs. Nevertheless, our results do provide new information on the time course over which these effects operate, and, we believe, impose some constraints on the physiological mechanisms involved.

Experiments 1 and 2 showed that the threshold difference between 500-pps and 3500-pps pulse trains was much smaller for ABI than for CI listeners, even at very short durations. Compelling evidence for an effect of a very fast-acting mechanism comes from a comparison between two conditions in experiment 2: two pulses separated by 2 ms and the same two pulses with this 2-ms gap filled with five 3500-pps pulses. The threshold difference between these two conditions was 3.8 dB for the CI users but only 0.46 dB for the ABI users. This result clearly rules out any explanation in terms of adaptation having time constants of the order of tens of milliseconds or longer, such as long-term or short-term adaptation. Hence, the substantial difference between electrical stimulation of the brainstem and of the auditory nerve is due to factors that operate within a few milliseconds. One potential factor is the residual depolarisation proposed by Middlebrooks (2004) to account for the effect of increasing pulse rate above about 1000 pps for CI listeners. A possible explanation for the lack of such an effect in ABI users could have been the absence of such charge interaction, such that the cell membrane returned to its resting potential after every pulse. Evidence against this explanation comes from the finding of experiment 3 that ABI listeners’ thresholds for a 3500-pps pulse train were lower for alternating polarity than for fixed polarity pulse trains, and that this difference was larger than at 500 pps. The effect was at least as large as for CI users, with a marginally significant difference in the opposite direction. Hence it seems that, at high pulse rates, successive pulses do interact at the level of the cell membrane, but that for some reason this does not result in a substantial threshold reduction.

One possible explanation for the small effect of pulse rate in ABI users is that the neurons near the stimulating ABI electrode are stimulated at a level that evokes their maximum possible firing rate, and that refractory effects, lasting a few milliseconds, prevent increases in pulse rate from leading to an increase in firing rate. This explanation would also require that, at high pulse rates, there are not substantial numbers of more distant neurons that can be recruited as pulse rate increases. In contrast, for CI stimulation, neurons might be firing lower down on their rate-level functions, a conjecture supported by psychophysical and modelling studies by McKay and McDermott (1998) who concluded that, at threshold, individual AN fibres fire with a probability of between 0.4 and 0.9 in response to every pulse. This would allow increases in charge summation, produced by increasing pulse rate, to produce additional action potentials in nearby fibres that had not been activated by the previous pulse.

The above explanation then raises the question of why increasing the current level does increase loudness for ABI users, even though increasing pulse rate does not reduce thresholds. The answer may be that brainstem neurons individually have small dynamic ranges (between threshold and saturation) so that, as current decays away from the stimulating electrode, there are only a few neurons that are not saturated, yet are sufficiently close to threshold to be able to increase their firing rate, or start to fire, when pulse rate is increased. In contrast, the increases in current level that cause loudness to grow may result from recruitment of neurons that are more distant from the stimulating electrode.

Although the different effect of pulse rate between ABI and CI users is apparent even at the very shortest durations, experiment 2 did show that, for some ABI users, the effect of pulse rate was slightly greater for the two shortest pulse trains than for longer durations. For these short durations, corresponding to NumPulse500 = 1 and 2, the duration of the 3500-pps pulse trains were 1.71 and 2.71 ms respectively. The threshold difference was reduced for values of NumPulse500 of 4 and higher, where the duration of the 3500 pps pulse train was 7.71 ms. Hence, it seems that some additional effect, with a time course of about 2.7 and 7.7 ms, reduced the influence of pulse rate on ABI users’ thresholds. One possibility is rapid adaptation. Zhang et al (2007) recorded spike-rate adaptation to electric pulse trains presented via an intra-cochlear electrode in the anesthetised cat and modelled the results using two adaptation time constants, of 8 and 80 ms, respectively. Neurons differed in the amount of adaptation, with fewer showing substantial amounts of adaptation for 250-pps pulse trains than for higher pulse rates. Hence, rapid rate-dependent adaptation can occur in response to electrical stimulation. Because the effect of pulse rate on thresholds did not change as duration was increased above 7.71 ms, there is no evidence that adaptation having longer time constant(s) play a role in the very small difference in thresholds at 500 vs 3500 pps for ABI users.

In summary, our investigation into why ABI users’ thresholds differ so little between 500 and 3500 pps rule out an explanation in terms of long-term adaptation and point to two fast-acting mechanisms. One of these is almost instantaneous and has an effect that can be observed for stimuli as short as 2 ms. We have argued that, although at 3500 pps successive pulses do interact at the level of the cell membrane, this does not lead to increases in the number of action potentials (relative to that occurring at lower pulse rates), possibly because all neurons that do respond are near the top of their dynamic range. A second mechanism, responsible for the fact the threshold difference between 500 and 3500-pps pulse trains decreases slightly with increases in duration up to 7.7 ms, has a time constant below about 10 ms and may reflect rapid adaptation.

#### Polarity

Experiment 4 showed that, like CI users, ABI users are more sensitive to QP-A than to QP-C pulses. We believe that there are two reasons for interpreting this finding as evidence that ABI users are more sensitive to anodic than to cathodic current. One is that the same finding occurs for CI users, for whom electrophysiological evidence confirms the greater sensitivity to anodic current. The other is that, in a QP pulse, the first and last phases each contain half the charge of the central phase and are therefore less likely to elicit neural activity. It must be acknowledged, however, that definitive proof should come from electrophysiological studies similar to those performed with CIs (Undurraga et al. 2010, 2013). We should also note that, although for QP pulses the effect of waveform polarity is similar for CIs and ABIs, the underlying mechanisms may not be the same for the two devices. For example, when a neuron contains multiple dendrites that are close to the stimulating electrode, anodic current can flow through the dendrites and soma, subsequently passing out through the membrane of the cell’s axon (Plonsey and Barr 2007). This mechanism will not operate for neurons of the AN, which consist of a cell body plus two axons (often termed the ‘peripheral process’ and ‘central axon’).

### Perceptual Effects of Changing Stimulus Level in CI and ABI Users

We have compared the effects of pulse rate, for two groups of listeners, using thresholds measured in dB. It should be pointed out that a given decibel change may not necessarily correspond to the same change in sensitivity (d′) for two groups of listeners. For example, if the slope of the underlying psychometric function were steeper for ABI than for CI listeners, then, although increasing pulse rate might produce a smaller threshold change for ABI than for CI users when measured in decibels, this dB change might correspond to the same change in d′. However, steeper psychometric functions would, presumably, also reduce the decibel change in thresholds obtained with other manipulations, such as increases in duration, that would be expected to increase sensitivity. In contrast, our results show that the effect of duration (Fig. 2B) was similar between groups, whereas the effect of rate (Fig. 1) was very different. For example, for a 500-pps pulse train, the effect of increasing duration over the entire range studied in experiment 2 was similar for ABI (3.7 dB) and CI (4.4 dB) users, whereas the effect of increasing the pulse rate to 3500 pps was much larger for the CI group than for the ABI group. Similarly, the effect of presenting a 3500-pps pulse train in fixed vs alternating polarity in experiment 3 was actually larger for the ABI than for the CI group, whereas the opposite was true for the effect of pulse rate.

### Practical Implications

Experiment 3 showed that ABI users’ thresholds for a 3500-pps pulse train were 1.25 dB lower when the leading polarity of the (symmetric) biphasic pulses alternated from pulse to pulse compared to when it was always cathodic. If a similar, or larger, effect occurred at supra-threshold levels, then this manipulation could increase the loudness that is achievable for a given current level. An important question is whether the manipulation would have a similar effect on the excitability of those neurons responsible for the unwanted, non-auditory sensations that can occur with brainstem stimulation. If not, then presenting stimuli in alternating polarity could provide a simple and easily implemented method of extending the number of electrodes that produce auditory but not non-auditory sensation. A similar point applies to the loudness differences that can be produced by stimulating the brainstem with asymmetric pulses in monopolar mode (experiment 4). We should note that, although the sizes of the effects observed are of the order of only one or two decibels, this corresponds, on average, to about one sixth of the dynamic range for the electrodes and patients studied here.

We believe that the most promising potential application may come from stimulation of the brainstem with asymmetric pulses in bipolar mode. Existing strategies use symmetric pulses in either monopolar or bipolar mode. Monopolar mode may provide a wide current spread. In bipolar mode, the current at each electrode is a polarity-inverted version of that at the other. With a symmetric biphasic pulse, then, each electrode will receive the ‘effective’ (anodic) phase, and so we would expect two loci of neural excitation, one near each electrode. The relative effectiveness at each locus will presumably depend on factors such as the neural survival close to each electrode, so a given channel will either excite two subsets of neurons (which may have different characteristic frequencies) or, if one electrode is in a ‘dead region’, a single subset but where the clinician does not know which one. Research with CI users has shown that this problem can be at least partly overcome by using asymmetric pulses (Macherey et al. 2010; Macherey et al. 2011) because only one electrode receives anodic current that is concentrated in time (this would correspond to the central portion of our QP pulses). The result is a more unimodal pattern of activation than is achievable with symmetric pulses. Indeed, the centre of gravity corresponding to the time-concentrated anodic phase at one electrode can be ‘pushed away’ from the other electrode. This occurs because, at sites between the two stimulating electrodes, the voltages arising from the opposite-polarity phases at the two electrodes are partially cancelled. As a result, when the electrode that receives the time-concentrated anodic phase of an asymmetric pulse is the most apical electrode of a CI, the resulting excitation pattern is more apical than can be achieved with symmetric pulses either in bipolar or monopolar mode. The fact that ABI users also show polarity sensitivity, albeit in monopolar mode, suggests that bipolar stimulation with asymmetric pulses may provide greater control over the locus of excitation produced by an ABI. A particular situation where this may be beneficial is where the position of the ABI is such that no electrodes produce auditory sensations with symmetric pulses; asymmetric pulses may allow one to focus excitation on sites close to or beyond the edge of the electrode array and to more selectively excite auditory neurons. Another possibility would be to use one electrode as the ‘return’ for all channels, such that this common electrode receives the cathodic time-concentrated part of an asymmetric pulse. This may provide the restricted current spread obtainable with bipolar stimulation, whilst producing substantial excitation near only one of the electrodes.
